# Communicating and disseminating research findings to study participants: Formative assessment of participant and researcher expectations and preferences

**DOI:** 10.1017/cts.2020.9

**Published:** 2020-01-20

**Authors:** Cathy L. Melvin, Jillian Harvey, Tara Pittman, Stephanie Gentilin, Dana Burshell, Teresa Kelechi

**Affiliations:** 1 College of Medicine, Medical University of South Carolina, Charleston, SC, USA; 2 College of Health Professions/Healthcare Leadership & Management, Medical University of South Carolina, Charleston, SC, USA; 3 South Carolina Clinical & Translational Research Institute (CTSA), Medical University of South Carolina, Charleston, SC, USA; 4 SOGI-SES Add Health Study Carolina Population Center, University of North Carolina at Chapel Hill, Chapel Hill, NC, USA; 5 College of Nursing, Medical University of South Carolina, Charleston, SC, USA

**Keywords:** Communication, dissemination, research findings, research participant preference, researcher preference

## Abstract

**Introduction:**

Translating research findings into practice requires understanding how to meet communication and dissemination needs and preferences of intended audiences including past research participants (PSPs) who want, but seldom receive, information on research findings during or after participating in research studies. Most researchers want to let others, including PSP, know about their findings but lack knowledge about how to effectively communicate findings to a lay audience.

**Methods:**

We designed a two-phase, mixed methods pilot study to understand experiences, expectations, concerns, preferences, and capacities of researchers and PSP in two age groups (adolescents/young adults (AYA) or older adults) and to test communication prototypes for sharing, receiving, and using information on research study findings.

**Principal Results:**

PSP and researchers agreed that sharing study findings should happen and that doing so could improve participant recruitment and enrollment, use of research findings to improve health and health-care delivery, and build community support for research. Some differences and similarities in communication preferences and message format were identified between PSP groups, reinforcing the best practice of customizing communication channel and messaging. Researchers wanted specific training and/or time and resources to help them prepare messages in formats to meet PSP needs and preferences but were unaware of resources to help them do so.

**Conclusions:**

Our findings offer insight into how to engage both PSP and researchers in the design and use of strategies to share research findings and highlight the need to develop services and support for researchers as they aim to bridge this translational barrier.

## Introduction

Since 2006, the National Institutes of Health Clinical and Translational Science Awards (CTSA) have aimed to advance science and translate knowledge into evidence that, if implemented, helps patients and providers make more informed decisions with the potential to improve health care and health outcomes [1,2]. This aim responded to calls by leaders in the fields of comparative effectiveness research, clinical trials, research ethics, and community engagement to assure that results of clinical trials were made available to participants and suggesting that providing participants with results both positive and negative should be the “ethical norm” [1,3]. Others noted that

on the surface, the concept of providing clinical trial results might seem straightforward but putting such a plan into action will be much more complicated. Communication with patients following participation in a clinical trial represents an important and often overlooked aspect of the patient-physician relationship. Careful exploration of this issue, both from the patient and clinician-researcher perspective, is warranted [4].

Authors also noted that no systematic approach to operationalizing this “ethical norm” existed and that evidence was lacking to describe either positive or negative outcomes of sharing clinical trial results with study participants and the community [4]. It was generally assumed, but not supported by research, that sharing would result in better patient–physician/researcher communication, improvement in patient care and satisfaction with care, better patient/participant understanding of clinical trials, and enhanced clinical trial accrual [4].

More recent literature informs these processes but also raises unresolved concerns about the communication and dissemination of research results. A 2008 narrative review of available data on the effects of communicating aggregate and individual research showed that

- *research participants* want aggregate and clinically significant individual study results made available to them despite the transient distress that communication of results sometimes elicits [3,5]. While differing in their preferences for specific channels of communication, they indicated that not sharing results fostered lack of participant trust in the health-care system, providers, and researchers [6] and an adverse impact on trial participation [5];
- *investigators* recognized their ethical obligation to at least offer to share research findings with recipients and the nonacademic community but differed on whether they should proactively re-contact participants, the type of results to be offered to participants, the need for clinical relevance before disclosure, and the stage at which research results should be offered [5]. They also reported not being well versed in communication and dissemination strategies known to be effective and not having funding sources to implement proven strategies for sharing with specific audiences [5];
- *members of the research enterprise* noted that while public opinion regarding participation in clinical trials is positive, clinical trial accrual remains low and that the failure to provide information about study results may be one of many factors negatively affecting accrual. They also called for better understanding of physician–researcher and patient attitudes and preferences and posit that development of effective mechanisms to share trial results with study participants should enhance patient–physician communication and improve clinical care and research processes [5].

A 2010 survey of CTSAs found that while professional and scientific audiences are currently the primary focus for communicating and disseminating research findings, it is equally vital to develop approaches for sharing research findings with other audiences, including individuals who participate in clinical trials [1,5]. Effective communication and dissemination strategies are documented in the literature [6,7], but most are designed to promote adoption of evidence-based interventions and lack of applicability to participants overall, especially to participants who are members of special populations and underrepresented minorities who have fewer opportunities to participate in research and whose preferences for receiving research findings are unknown [7].

Researchers often have limited exposure to methods that offer them guidance in communicating and disseminating study findings in ways likely to improve awareness, adoption, and use of their findings [7]. Researchers also lack expertise in using communication channels such as traditional journalism platforms, live or face-to-face events such as public festivals, lectures, and panels, and online interactions [8]. Few strategies provide guidance for researchers about how to develop communications that are patient-centered, contain plain language, create awareness of the influence of findings on participant or population health, and increase the likelihood of enrollment in future studies.

Consequently, researchers often rely on traditional methods (e.g., presentations at scientific meetings and publication of study findings in peer-reviewed journals) despite evidence suggesting their limited reach and/or impact among professional/scientific and/or lay audiences [9,10].

Input from stakeholders can enhance our understanding of how to assure that participants will receive understandable, useful information about research findings and, as appropriate, interpret and use this information to inform their decisions about changing health behaviors, interacting with their health-care providers, enrolling in future research studies, sharing their study experiences with others, or recommending to others that they participate in studies.

### Purpose and Goal

This pilot project was undertaken to address issues cited above and in response to expressed concerns of community members in our area about not receiving information on research studies in which they participated. The project design, a two-phase, mixed methods pilot study, was informed by their subsequent participation in a committee of community-academic representatives to determine possible options for improving the communication and dissemination of study results to both study participants and the community at large.

Our goals were to understand the experiences, expectations, concerns, preferences, and capacities of researchers and past research participants (PSP) in two age groups (adolescents/young adults (AYA) aged 15–25 years and older adults aged 50 years or older) and to test communication prototypes for sharing, receiving, and using information on research study findings. Our long-term objectives are to stimulate new, interdisciplinary collaborative research and to develop resources to meet PSP and researcher needs.

## Methods

### Overview

**Fig. 1.**
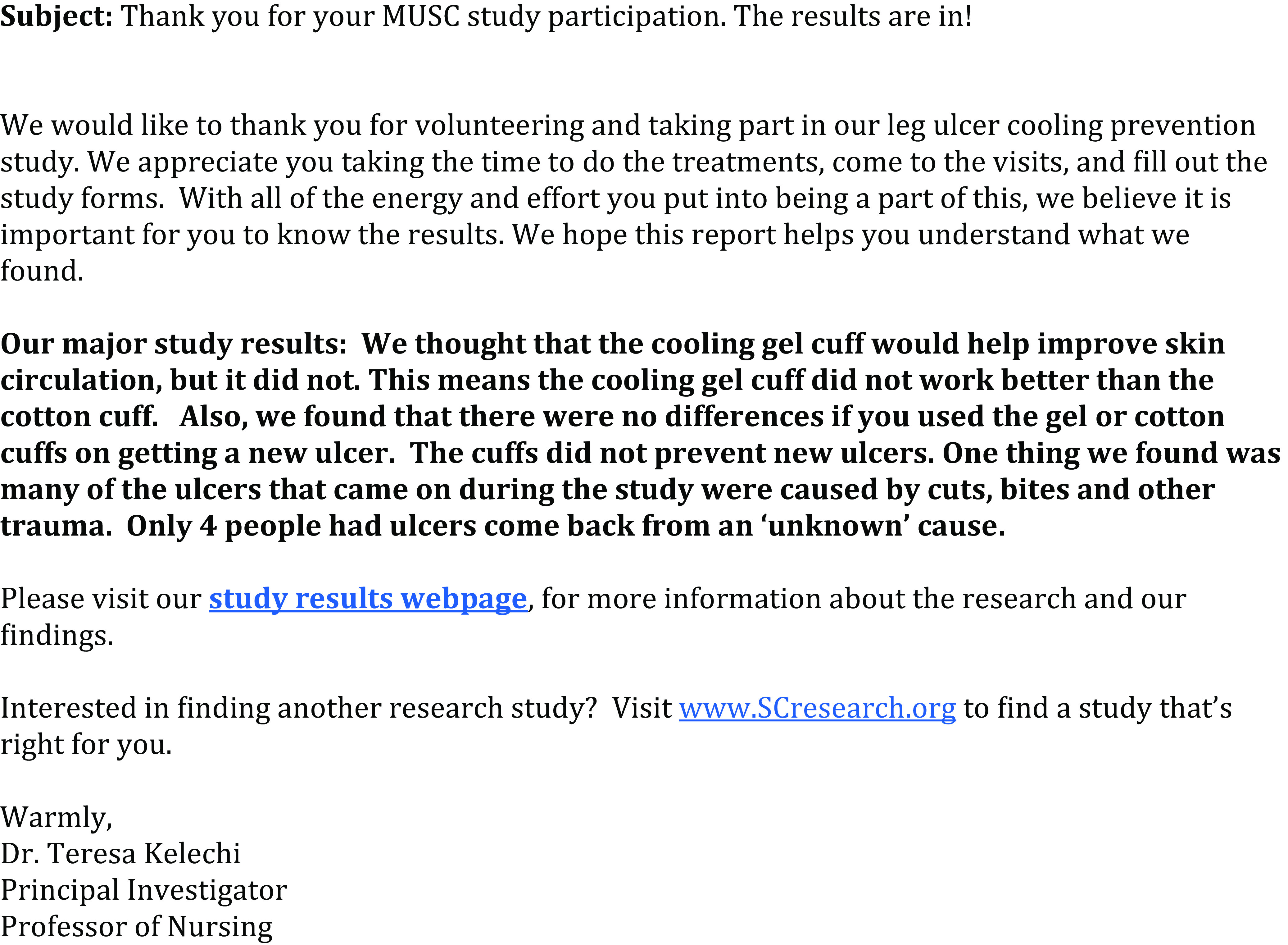
Prototype 1: study results email prototype. MUSC, Medical University of South Carolina.

**Fig. 2.**
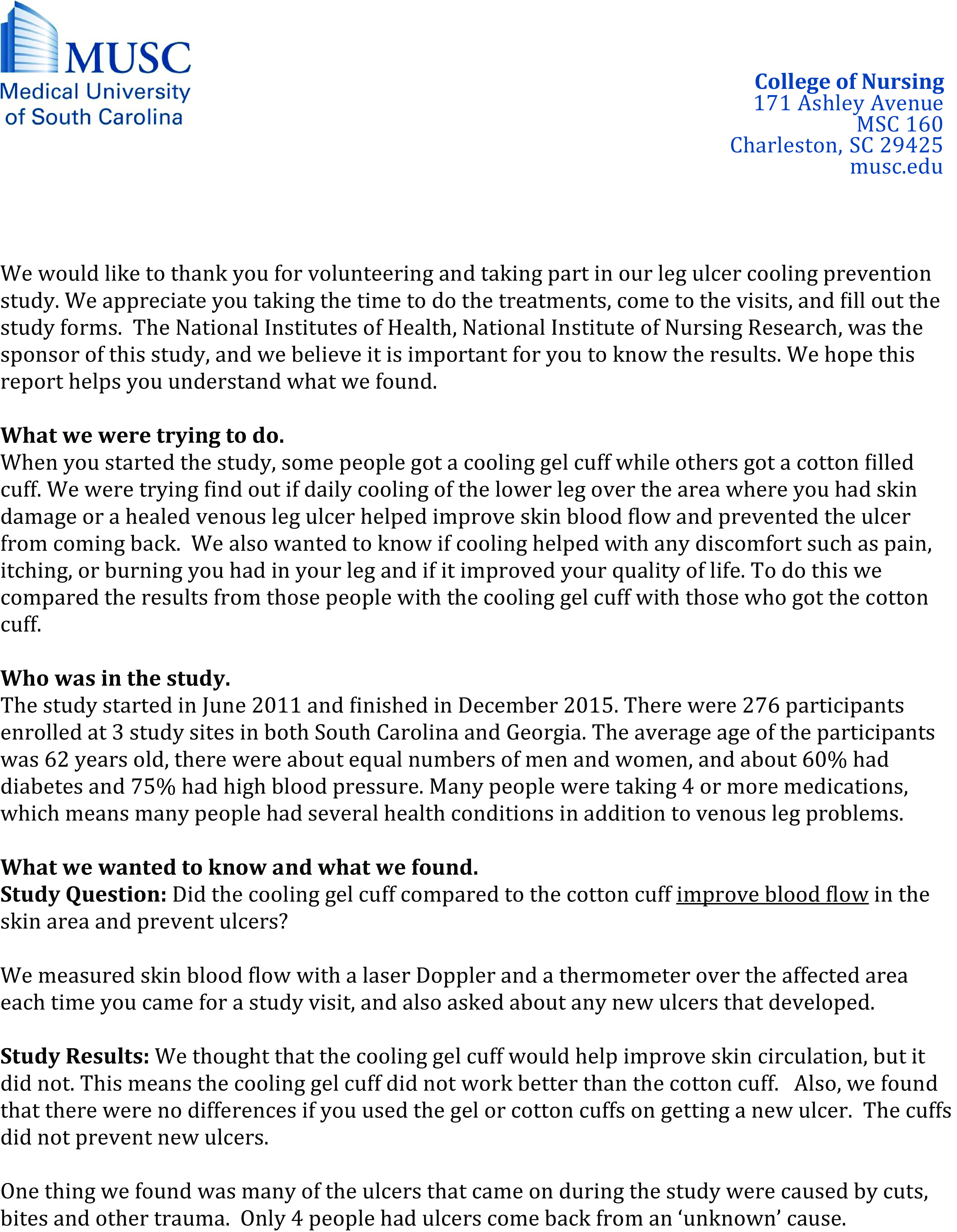
Prototype 2: study results letter prototype.

**Fig. 3.**
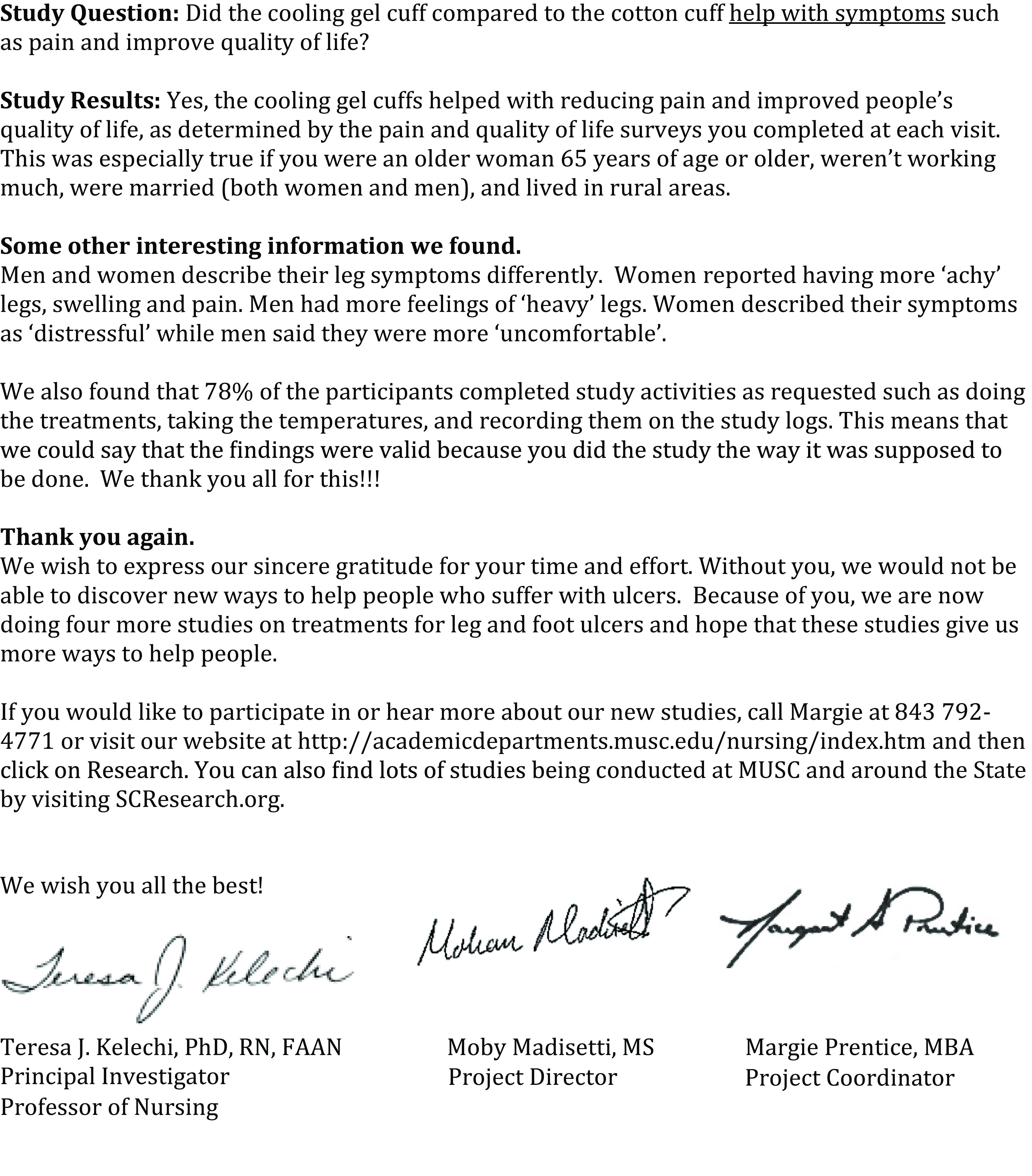
Prototype 3: study results MailChimp prototypes 1 and 2. MUSC, Medical University of South Carolina.

This study was conducted in an academic medical center located in south-eastern South Carolina. Phase one consisted of surveying PSP and researchers. In phase two, in-person focus groups were conducted among PSP completing the survey and one-on-one interviews were conducted among researchers. Participants in either the interviews or focus groups responded to a set of questions from a discussion guide developed by the study team and reviewed three prototypes for communicating and disseminating study results developed by the study team in response to PSP and researcher survey responses: a study results letter, a study results email, and a web-based communication – Mail Chimp (Figs. 1–3).

### PSP and researcher surveys

A 42-item survey questionnaire representing seven domains was developed by a multidisciplinary team of clinicians, researchers, and PSP that evaluated the questions for content, ease of understanding, usefulness, and comprehensiveness [11]. Project principal investigators reviewed questions for content and clarity [11]. The PSP and researcher surveys contained screening and demographic questions to determine participant eligibility and participant characteristics. The PSP survey assessed prior experience with research, receipt of study information from the research team, intention to participate in future research, and preferences and opinions about receipt of information about study findings and next steps. Specific questions for PSP elicited their preferences for communication channels such as phone call, email, social or mass media, and public forum and included channels unique to South Carolina, such as billboards. PSP were asked to rank their preferences and experiences regarding receipt of study results using a Likert scale with the following measurements: “not at all interested” (0), “not very interested” (1), “neutral” (3), “somewhat interested” (3), and “very interested” (4).

The researcher survey contained questions about researcher decisions, plans, and actions regarding communication and dissemination of research results for a recently completed study. Items included knowledge and opinions about how to communicate and disseminate research findings, resources used and needed to develop communication strategies, and awareness and use of dissemination channels, message development, and presentation format.

A research team member administered the survey to PSP and researchers either in person or via phone. Researchers could also complete the survey online through Research Electronic Data Capture (REDCap©).

### Focus groups and discussion guide content

The PSP focus group discussion guide contained questions to assess participants’ past experiences with receiving information about research findings; identify participant preferences for receiving research findings whether negative, positive, or equivocal; gather information to improve communication of research results back to participants; assess participant intention to enroll in future research studies, to share their study experiences with others, and to refer others to our institution for study participation; and provide comments and suggestions on prototypes developed for communication and dissemination of study results. Five AYA participated in one focus group, and 11 older adults participated in one focus group. Focus groups were conducted in an off-campus location with convenient parking and at times convenient for participants. Snacks and beverages were provided.

The researcher interview guide was designed to understand researchers’ perspectives on communicating and disseminating research findings to participants; explore past experiences, if any, of researchers with communication and dissemination of research findings to study participants; document any approaches researchers may have used or intend to use to communicate and disseminate research findings to study participants; assess researcher expectations of benefits associated with sharing findings with participants, as well as, perceived and actual barriers to sharing findings; and provide comments and suggestions on prototypes developed for communication and dissemination of study results.

### Prototype materials

Three prototypes were presented to focus group participants and included (1) a formal letter on hospital letterhead designed to be delivered by standard mail, describing the purpose and findings of a fictional study and thanking the individual for his/her participation, (2) a text-only email including a brief thank you and a summary of major findings with a link to a study website for more information, and (3) an email formatted like a newsletter with detailed information on study purpose, method, and findings with graphics to help convey results. A mock study website was shown and included information about study background, purpose, methods, results, as well as, links to other research and health resources. Prototypes were presented either in paper or PowerPoint format during the focus groups and explained by a study team member who then elicited participant input using the focus group guide. Researchers also reviewed and commented on prototype content and format in one-on-one interviews with a study team member.

## Protection of Human Subjects

The study protocol (No. Pro00067659) was submitted to and approved by the Institutional Review Board at the Medical University of South Carolina in 2017. PSP (or the caretakers for PSP under age 18), and researchers provided verbal informed consent prior to completing the survey or participating in either a focus group or interview. Participants received a verbal introduction prior to participating in each phase.

## Recruitment and Interview Procedures

### Past study participants

A study team member reviewed study participant logs from five recently completed studies at our institution involving AYA or older adults to identify individuals who provided consent for contact regarding future studies. Subsequent PSP recruitment efforts based on these searches were consistent with previous contact preferences recorded in each study participant’s consent indicating desire to be re-contacted. The primary modes of contact were phone/SMS and email.

Efforts to recruit other PSP were made through placement of flyers in frequented public locations such as coffee shops, recreation complexes, and college campuses and through social media, Yammer, and newsletters. ResearchMatch, a web-based recruitment tool, was used to alert its subscribers about the study. Potential participants reached by these methods contacted our study team to learn more about the study, and if interested and pre-screened eligible, volunteered and were consented for the study. PSP completing the survey indicated willingness to share experiences with the study team in a focus group and were re-contacted to participate in focus groups.

### Researcher recruitment

Researchers were identified through informal outreach by study investigators and staff, a flyer distributed on campus, use of Yammer and other institutional social media platforms, and internal electronic newsletters. Researchers responding to these recruitment efforts were invited to participate in the researcher survey and/or interview.

### Incentives for participation

Researchers and PSP received a $25 gift card for completing the survey and $75 for completing the interview (researcher) or focus group (PSP) (up to $100 per researcher or PSP).

## Analysis

Data tables displaying demographic and other data from the PSP surveys (Table 1) were prepared from the REDCap© database and responses reported as number and percent of respondents choosing each response option.

**Table 1.** Post study participant (PSP) characteristics by Adolescents/Young Adults (AYA), Older Adults, and ALL (All participants regardless of age)

Characteristics	AYA (age 15–24.99 years) (*n* = 15)	Older adult (age 50 years or more) (*n* = 33)	ALL (*n* = 48)
Race			
Black African American	2 (13%)	8 (24%)	10 (21%)
White	12 (80%)	25 (76%)	37 (77%)
More than one race	1 (7%)	--	1 (2%)
Gender			
Female	12 (80%)	25 (76%)	37 (77%)
Male	3 (20%)	8 (24%)	11 (23%)
Education			
Grade 9–12	-	-	-
High-school graduate	2 (13%)	8 (24%)	10 (21%)
Some college	2 (13%)	12 (36%)	14 (29%)
Associate degree	-	1 (3%)	1 (2%)
Bachelor’s degree	9 (60%)	7 (21%)	16 (33%)
Master’s degree	1 (7%)	5 (16%)	6 (13%)
Professional degree	1 (7%)	-	1 (2%)
Ethnicity			
Not Hispanic/Latino	14 (93%)	32 (97%)	46 (96%)
Hispanic Latino	1 (7%)	1 (3%)	2 (4%)

Age mean (SD) = 49.7 (18.6).

Focus group and researcher interview data were recorded (either via audio recording and/or notes taken by research staff) and analyzed via a general inductive qualitative approach, a method appropriate for program evaluation studies and aimed at condensing large amounts of textual data into frameworks that describe the underlying process and experiences under study [12]. Data were analyzed by our team’s qualitative expert who read the textual data multiple times, developed a coding scheme to identify themes in the textual data, and used group consensus methods with other team members to identify unique, key themes.

## Results

### PSP Survey

Sixty-one of sixty-five PSP who volunteered to participate in the PSP survey were screened eligible, fifty were consented, and forty-eight completed the survey questionnaire. Of the 48 PSP completing the survey, 15 (32%) were AYA and 33 (68%) older adults. The mean age of survey respondents was 49.7 years, 23.5 for AYA, and 61.6 for older adults. Survey respondents were predominantly White, non-Hispanic/Latino, female, and with some college or a college degree (Table 1). The percentage of participants in each group never or rarely needing any help with reading/interpreting written materials was above 93% in both groups.

Over 90% of PSP responded that they would participate in another research study, and more than 75% of PSP indicated that study participants should know about study results. Most (68.8%) respondents indicated that they *did not receive any communications from study staff after they finished a study*.

PSP preferences for communication channel are summarized in Table 2 and based on responses to the question “How do you want to receive information?.” Both AYA and older adults agree or completely agree that they prefer email to other communication channels and that billboards did not apply to them. Older adult preferences for communication channels as indicated by agreeing or completely agreeing were in ranked order of highest to lowest: use of mailed letters/postcards, newsletter, and phone. A majority (over 50%) of older adults completely disagreed or disagreed on texting and social media as options and had only slight preference for mass media, public forum, and wellness fairs or expos.

**Table 2.** Communication preference by group: AYA*, older adult**, and ALL (*n* = 48)

Communication format	Completely disagree	Disagree	Neutral	Agree	Completely agree	Don’t know	Not applicable
Phone							
AYA	4 (26.7)	3 (20)	6 (40.0)	1 (6.7)	1 (6.7)	-	-
Older adult	10 (30.3)	1 (3)	6 (18.2)	2 (6.1)	14 (42.4)	-	-
ALL	14 (29.2)	4 (8.3)	12 (25.0)	3 (9.1)	15 (31.3)	-	-
Mailed letters, postcards							
AYA	5 (33.3)	4 (26.7)	2 (13.3)	2 (13.3)	2 (13.3)	-	-
Older adult	3 (9.1)	2 (6.1)	5 (15.2)	7 (21.2)	16 (48.5)	-	-
ALL	8 (16.7)	6 (12.5)	7 (14.6)	9 (18.8)	18 (37.5)	-	-
Email							
AYA	-	-	-	3 (20)	12 (80)	-	-
Older adult	5 (15.2)	1 (3.0)	2 (6.1)	2 (6.1)	21 (63.6)	-	-
ALL	5 (10.4)	1 (2.1)	2 (4.2)	5 (10.4)	33 (68.8)	-	-
Texting							
AYA	5 (33.3)	2 (13.3)	2 (13.3)	4 (26.7)	2 (13.3)	-	-
Older adult	17 (51.5)	1 (3.0)	4 (12.1)	3 (9.1)	4 (12.1)	-	-
ALL	22 (45.8)	3 (6.3)	6 (12.5)	7 (14.6)	6 (12.5)	-	-
Newsletter							
AYA	5 (33.3)	3 (20.0)	4 (26.7)	1 (6.7)	2 (13.3)	-	-
Older adult	4 (12.1)	2 (6.1)	8 (24.2)	6 (18.2)	13 (39.4)	-	-
ALL	9 (18.8)	5 (10.4)	12(25)	7 (14.6)	15 (31.3)	-	-
Social media							
AYA	5 (33.3)	5 (33.3)	4 (26.7)	-	1 (6.7)	-	-
Older adult	20 (60.6)	-	4 (12.1)	1 (3.0)	6 (21.2)	-	-
ALL	25 (52.1)	5 (10.4)	8 (16.7)	1 (2.1)	7 (14.6)	-	-
Mass media							
AYA	3 (20.0)	6 (40.0)	6 (40.0)	-	-	-	
Older adult	14 (42.4)	2 (6.1)	7 (21.2)	4 (12.1)	6 (18.2)	-	
ALL	17 (35.4)	8 (16.7)	13 (27.1)	4 (8.3)	6 (12.5)	-	
Public forum							
AYA	5 (33.3)	2 (13.3)	6 (40.0)	1 (6.7)	1 (6.7)		
Older adult	12 (36.4)	4 (12.1)	5 (15.2)	6 (18.2)	6 (18.2)		
ALL	17 (35.4)	6 (12.5)	11 (22.9)	7 (14.6)	7 (14.6)		
Wellness fair/expo							
AYA	4 (26.7)	1 (6.7)	5 (33.3)	5 (33.3)	-	-	-
Older adult	12 (36.4)	3 (9.1)	9 (27.3)	2 (6.1)	7 (21.2)		
ALL	16 (33.3)	4 (8.3)	14 (29.4)	7 (14.6)	7 (14.6)	-	-
Other (billboard)							
AYA	-	-	-	-	1 (1.67)	3 (20.0)	11 (73.3)
Older adult	2 (6.1)	-	1(3.0)	-	1 (3.0)	8 (3)	-
ALL	2 (14.2)	-	-	1 (2.1)	1 (2.1)	4 (8.3)	39 (81.3)

ALL, total per column.

* AYA: adolescent/young adult (age 15–24.99 years) (*n* = 15).

** Older adult (age 50 years or more) (*n* = 33).

While AYA preferred email over all other options, they completely disagreed/disagreed with mailed letters/postcards, social media, and mass media options.

When communication formats were ranked overall by each group and by both groups combined, the ranking from most to least preferred was written materials, opportunities to interact with study teams and ask questions, visual charts, graphs, pictures, and videos, audios, and podcasts.

### PSP Focus Groups

*PSP want to receive and share information on study findings* for studies in which he/she participated. Furthermore, participants stated their desire to share study results across social networks and highlighted opportunities to share communicated study results with their health-care providers, family members, friends, and other acquaintances with similar medical conditions.

Because of the things I was in a study for, it’s a condition I knew three other people who had the same condition, so as soon as it worked for me, I put the word out, this is great stuff.

I would forward the email with the link, this is where you can go to also get in on this study, or I’d also tell them, you know, for me, like the medication. Here’s the medication. Here’s the name of it. Tell your doctor.

I would definitely share. I’d just tell everyone without a doubt. Right when I get home, as soon as I walk in the door, and say Renee-that’s my daughter-I’ve got to tell you this.

Communication of study information could happen through several channels including social media, verbal communication, sharing of written documents, and forwarding emails containing a range of content in a range of formats (e.g., reports and pamphlets).

Word of mouth and I have no shame in saying I had head to toe psoriasis, and I used the drug being studied, and so I would just go to people, hey, look.

So, if you had it in paper form, like a pamphlet or something, yeah I’d pass it on to them.

*PSP prefer clear, simple messaging* and highlighted multiple, preferred communication modalities for receiving information on study findings including emails, letters, newsletters, social media, and websites.

The wording is really simple, which I like. It’s to the point and clear.

I really like the bullet points, because it’s quick and to the point.

I think the [long] paragraphs-you get lost, especially when you are reading on your phone.

They indicated a clear preference for colorful, simple, easy to read communication. PSP also expressed some concern about difficulty opening emails with pictures and dislike lengthy written text. “I don’t read long emails. I tend to delete them”

PSP indicated some confusion about common research language. For example, one participant indicated that using the word “estimate” indicates the research findings were an approximation, “When I hear those words, I just think you’re guessing, estimate, you know? It sounds like an estimate, not a definite answer.”

### Researcher Survey

Twenty-three of thirty-two researchers volunteered to participate in the researcher survey, were screened eligible, and two declined to participate, resulting in 19 who provided consent to participate and completed the survey. The mean age of survey respondents was 51.8 years. Respondents were predominantly White, non-Hispanic/Latino, and female, and all were holders of either a professional school degree or a doctoral degree. When asked if it is important to inform study participants of study results, 94.8% of responding researchers agreed that it was extremely important or important. Most researchers have disseminated findings to study participants or plan to disseminate findings.

Researchers listed a variety of reasons for their rating of the importance of informing study participants of study results including “to promote feelings of inclusion by participants and other community members”, “maintaining participant interest and engagement in the subject study and in research generally”, “allowing participants to benefit somewhat from their participation in research and especially if personal health data are collected”, “increasing transparency and opportunities for learning”, and “helping in understanding the impact of the research on the health issue under study”.

Some researchers view sharing study findings as an “ethical responsibility and/or a tenet of volunteerism for a research study”. For example, “if we (researchers) are obligated to inform participants about anything that comes up during the conduct of the study, we should feel compelled to equally give the results at the end of the study”.

One researcher “thought it a good idea to ask participants if they would like an overview of findings at the end of the study that they could share with others who would like to see the information”.

Two researchers said that sharing research results “depends on the study” and that providing “general findings to the participants” might be “sufficient for a treatment outcome study”.

Researchers indicated that despite their willingness to share study results, they face resource challenges such as a lack of funding and/or staff to support communication and dissemination activities and need assistance in developing these materials. One researcher remarked “I would really like to learn what are (sic) the best ways to share research findings. I am truly ignorant about this other than what I have casually observed. I would enjoy attending a workshop on the topic with suggested templates and communication strategies that work best” and that this survey “reminds me how important this is and it is promising that our CTSA seems to plan to take this on and help researchers with this important study element.”

Another researcher commented on a list of potential types of assistance that could be made available to assist with communicating and disseminating results, that “Training on developing lay friendly messaging is especially critically important and would translate across so many different aspects of what we do, not just dissemination of findings. But I’ve noticed that it is a skill that very few people have, and some people never can seem to develop. For that reason, I find as a principal investigator that I am spending a lot of my time working on these types of materials when I’d really prefer research assistant level folks having the ability to get me 99% of the way there.”

Most researchers indicated that they provide participants with personal tests or assessments taken from the study (60% *n* = 6) and final study results (72.7%, *n* = 8) but no other information such as recruitment and retention updates, interim updates or results, information on the impact of the study on either the health topic of the study or the community, information on other studies or provide tips and resources related to the health topic and self-help. Sixty percent (*n* = 6) of researcher respondents indicated sharing planned next steps for the study team and information on how the study results would be used.

When asked about how they communicated results, phone calls were mentioned most frequently followed by newsletters, email, webpages, public forums, journal article, mailed letter or postcard, mass media, wellness fairs/expos, texting, or social media.

Researchers used a variety of communication formats to communicate with study participants. Written descriptions of study findings were most frequently reported followed by visual depictions, opportunities to interact with study staff and ask questions or provide feedback, and videos/audio/podcasts.

Seventy-three percent of researchers reported that they made efforts to make study findings information available to those with low levels of literacy, health literacy, or other possible limitations such as non-English-speaking populations.

In open-ended responses, most researchers reported wanting to increase their awareness and use of on-campus training and other resources to support communication and dissemination of study results, including how to get resources and budgets to support their use.

### Researcher Interviews

One-on-one interviews with researchers identified two themes.

#### Researchers may struggle to see the utility of communicating small findings

Some researchers indicated hesitancy in communicating preliminary findings, findings from small studies, or highly summarized information. In addition, in comparison to research participants, researchers seemed to place a higher value on specific details of the study.

“I probably wouldn’t put it up [on social media] until the actual manuscript was out with the graphs and the figures, because I think that’s what people ultimately would be interested in.”

#### Researchers face resource and time limitations in communication and dissemination of study findings

Researchers expressed interest in communicating research results to study participants. However, they highlighted several challenges including difficulties in tracking current email and physical addresses for participants; compliance with literacy and visual impairment regulations; and the number of products already required in research that consume a considerable amount of a research team’s time. Researchers expressed a desire to have additional resources and templates to facilitate sharing study findings. According to one respondent, “For every grant there is (sic) 4-10 papers and 3-5 presentations, already doing 10-20 products.” Researchers do not want to “reinvent the wheel” and would like to pull from existing papers and presentations on how to share with participants and have boilerplate, writing templates, and other logistical information available for their use.

Researchers would also like training in the form of lunch-n-learns, podcasts, or easily accessible online tools on how to develop materials and approaches. Researchers are interested in understanding the “do’s and don’ts” of communicating and disseminating study findings and any regulatory requirements that should be considered when communicating with research participants following a completed study. For example, one researcher asked, “From beginning to end – the do’s and don’ts – are stamps allowed as a direct cost? or can indirect costs include paper for printing newsletters, how about designing a website, a checklist for pulling together a newsletter?”

## Discussion

The purpose of this pilot study was to explore the current experiences, expectations, concerns, preferences, and capacities of PSP including youth/young adult and older adult populations and researchers for sharing, receiving, and using information on research study findings. PSP and researchers agreed, as shown in earlier work [3,5], that sharing information upon study completion with participants was something that should be done and that had value for both PSP and researchers. As in prior studies [3,5], both groups also agreed that sharing study findings could improve ancillary outcomes such as participant recruitment and enrollment, use of research findings to improve health and health-care delivery, and build overall community support for research. In addition, communicating results acknowledges study participants’ contributions to research, a principle firmly rooted in respect for treating participants as not merely a means to further scientific investigation [5].

The majority of PSP indicated that they did not receive research findings from studies they participated in, that they would like to receive such information, and that they preferred specific communication methods for receipt of this information such as email and phone calls. While our sample was small, we did identify preferences for communication channels and for message format. Some differences and similarities in preferences for communication channels and message format were identified between AYA and older adults, thus reinforcing the best practice of customizing communication channel and messaging to each specific group. However, the preference for email and the similar rank ordering of messaging formats suggest that there are some overall communication preferences that may apply to most populations of PSP. It remains unclear whether participants prefer individual or aggregate results of study findings and depends on the type of study, for example, individual results of genotypes versus aggregate results of epidemiological studies [13]. A study by Miller et al suggests that the impact of receiving aggregate results, whether clinically relevant or not, may equal that of receiving individual results [14]. Further investigation warrants evaluation of whether, when, and how researchers should communicate types of results to study participants, considering multiple demographics of the populations such as age and ethnicity on preferences.

While researchers acknowledged that PSP would like to hear from them regarding research results and that they wanted to meet this expectation, they indicated needing specific training and/or time and resources to provide this information to PSP in a way that meets PSP needs and preferences. Costs associated with producing reports of findings were a concern of researchers in our study, similar to findings from a study conducted by Di Blasi and colleagues in which 15% (8 of 53 investigators) indicated that they wanted to avoid extra costs associated with the conduct of their studies and extra administrative work [15]. In this same study, the major reason for not informing participants about study results was that forty percent of investigators never considered this option. Researchers were unaware of resources available on existing platforms at their home institution or elsewhere to help them with communication and dissemination efforts [10].

### Addressing Barriers to Implementation

Information from academic and other organizations on how to best communicate research findings in plain language is available and could be shared with researchers and their teams. The Cochrane Collaborative [16], the Centers for Disease Control and Prevention [17], and the Patient-Centered Outcomes Research Institute [18] have resources to help researchers develop plain language summaries using proven approaches to overcome literacy and other issues that limit participant access to study findings. Some academic institutions have electronic systems in place to confidentially share templated laboratory and other personal study information with participants and, if appropriate, with their health-care providers.

## Limitations

Findings from the study are limited by several study and respondent characteristics. The sample was drawn from research records at one university engaging in research in a relatively defined geographic area and among two special populations: AYA and older adults. As such, participants were not representative of either the general population in the area, the population of PSP or researchers available in the area, or the racial and ethnic diversity of potential and/or actual participants in the geographic area. The small number of researcher participants did not represent the pool of researchers at the university, and the research studies from which participants were drawn were not representative of the broad range of clinical and translational research undertaken by our institution or within the geographic community it serves. The number of survey and focus group participants was insufficient to allow robust analysis of findings specific to participants’ race, ethnicity, gender, or membership in the target age groups of AYA or older adult. However, these data will inform a future trial with adequate representations from underrepresented and special population groups.

Since all PSP had participated in research, they may have been biased in favor of wanting to know more about study results and/or supportive/nonsupportive of the method of communication/dissemination they were exposed to through their participation in these studies.

## Conclusions

Our findings provide information from PSP and researchers on their expectations about sharing study findings, preferences for how to communicate and disseminate study findings, and need for greater assistance in removing roadblocks to using proven communication and dissemination approaches. This information illustrates the potential to engage both PSP and researchers in the design and use of communication and dissemination strategies and materials to share research findings, engage in efforts to more broadly disseminate research findings, and inform our understanding of how to interpret and communicate research findings for members of special population groups. While several initial prototypes were developed in response to this feedback and shared for review by participants in this study, future research will focus on finalizing and testing specific communication and dissemination prototypes aimed at these special population groups.

Findings from our study support a major goal of the National Center for Advancing Translational Science Recruitment Innovation Center to engage and collaborate with patients and their communities to advance translation science. In response to the increased awareness of the importance of sharing results with study participants or the general public, a template for dissemination of research results is available in the Recruitment and Retention Toolbox through the CTSA Trial Innovation Network (TIN: trialinnovationnetwork.org). We believe that our findings will inform resources for use in special populations through collaborations within the TIN.
